# Honey bee microbiome associated with different hive and sample types over a honey production season

**DOI:** 10.1371/journal.pone.0223834

**Published:** 2019-11-08

**Authors:** Sladjana Subotic, Andrew M. Boddicker, Vy M. Nguyen, James Rivers, Christy E. Briles, Annika C. Mosier

**Affiliations:** 1 Department of Integrative Biology, University of Colorado, Denver, Colorado, United States of America; 2 Department of Geography and Environmental Sciences, University of Colorado, Denver, Colorado, United States of America; University of Illinois, UNITED STATES

## Abstract

Western honey bees (*Apis mellifera*) are important pollinators in natural and agricultural ecosystems, and yet are in significant decline due to several factors including parasites, pathogens, pesticides, and habitat loss. A new beehive construction called the Flow^TM^ hive was developed in 2015 to allow honey to be harvested directly from the hive without opening it, resulting in an apparent decrease in stress to the bees. Here, we compared the Flow and traditional Langstroth hive constructions to determine if there were any significant differences in the bee microbiome. The bee-associated bacterial communities did not differ between hive constructions and varied only slightly over the course of a honey production season. Samples were dominated by taxa belonging to the *Lactobacillus*, *Bifidobacterium*, *Bartonella*, *Snodgrassella*, *Gilliamella*, and *Frischella* genera, as observed in previous studies. The top ten most abundant taxa made up the majority of the sequence data; however, many low abundance organisms were persistent across the majority of samples regardless of sampling time or hive type. We additionally compared different preparations of whole bee and dissected bee samples to elaborate on previous bee microbiome research. We found that bacterial sequences were overwhelming derived from the bee guts, and microbes on the bee surfaces (including pollen) contributed little to the overall microbiome of whole bees. Overall, the results indicate that different hive constructions and associated disturbance levels do not influence the bee gut microbiome, which has broader implications for supporting hive health.

## Introduction

The western honey bee (*Apis mellifera*) plays a critical role in maintaining the health of natural and agricultural ecosystems [1]. Honey bees are one of the most widely distributed pollinators and most frequent floral visitors amongst animal pollinators [1]. More than 90 different commercially grown plants harvested for food in North America rely on the honey bee for pollination [2]. Honey bee pollination contributes billions of dollars annually to the United States economy, with a far greater economic impact on the global scale [3, 4]. However, recent drastic decreases in honey bee populations associated with Colony Collapse Disorder (CCD) threaten to destabilize plant reproduction in many land-based ecosystems [5–7].

*A*. *mellifera* in managed artificial hives have substantially weakened immune function compared to wild hives [8–10]. Immune function is likely impacted by environmental stressors including reduced access to foraging, pesticide exposure, antibiotic exposure, and controlled migration that occur during hive management [7, 11–13]. Compromised immune function can leave *A*. *mellifera* vulnerable to opportunistic infections that impact rates of individual and colony survival, such as infections from viruses (e.g., *Picornavirales*), protozoa (e.g., *Crithidia*), bacteria (e.g., *Melissococcus plutonius*), and fungi (e.g., *Ascosphaera apis*) [7, 14–16].

Environmental stressors have been shown to alter the indigenous gut microbiota in *A*. *mellifera* [13]. Diseased bees often show dysbiosis, with increases or decreases in the dominant microbial taxa or occurrence of transient bacteria not normally associated with the host [17]. CCD-impacted hives have shown decreases in some taxa (e.g., *Bifidobacterium*), as well as increases in the abundance of other important taxa (e.g., *Firmicutes*) [15]. When dysbiosis occurs, deviations to diversity and relative abundance patterns of the microbiome can have devastating health effects [13] and *A*. *mellifera* can become more susceptible to infection.

Stressors in managed hives can range from the frequency of opening hives to the materials used to create an enclosed space for the bees. In Spring 2015, a new beehive construction called the Flow hive [18] was developed to reduce stress to the colony compared to long-standing Langstroth hives, which are structurally mostly wood with thin plastic waxed foundations that the bees build wax cells, store nectar, and cap them once the nectar reaches the right water content level (i.e., honey). The Flow frame design consists of partially formed hexagonal cells; the bees complete the cells by coating them with wax prior to filling with honey and capping. The plastic cells can be split by the beekeeper when the honey is ready without opening the hive and with little disturbance, and presumably stress, to the colony.

Here we compare the *A*. *mellifera* microbiome from traditional Langstroth hives and the new Flow frames using deep amplicon sequencing of the bacterial 16S rRNA gene. We evaluate microbial community structure and diversity among frame types and over a honey production season. We also compare the bacterial community associated with the *A*. *mellifera* gut to the whole bee microbiome (including microbes within and on the bees). This study extends our understanding of the common and rare microbial taxa associated with bees living in different hive types and sets the stage for future work aimed at utilizing microbial community signatures as indicators of colony stability, health, and disease state.

## Materials and methods

### Experimental design

Twenty bee hives were housed at the 13-acre Five Fridges Farm in Wheatridge, CO. Permission to conduct the study on this site was granted through a memorandum of understanding (MOU) between Five Fridges Farm and the University of Colorado, Denver. The field studies did not involve endangered or protected species. The farm is in a suburban setting surrounded by apartment buildings, houses and schools. The hive comparison involved ten traditional Langstroth and ten Flow hives. The bottom brood boxes all contained traditional Langstroth frames and parts (as recommended by Flow). The supers (a box on top of the brood where honey is harvested) contained either the traditional Langstroth frames (n = 10 hives; plastic waxed foundation in a wood frame) or the plastic Flow frames (n = 10 hives). Four Flow frames were placed into each super and the empty space on either side was filled with traditional frames. An equal volume of wooden frames (six) were placed in each of the Langstroth supers. Italian western honey bees were acquired from Apis Hive Company in Grand Junction, CO and the hives were colonized in early May 2016. Brood boxes were inspected for measures of health approximately once a month, including pest infestations, queen bee condition, and honey production. Honey harvesting resulted in different disturbance levels due to inherent structural differences between traditional and Flow hives (i.e., traditional frames were removed from the hive and scraped of wax and honey, whereas Flow preformed plastic cells were split but the structure remained resulting in presumably lower energetic costs for the bees).

### Bee sampling

For microbial analysis bees were sampled over the honey production season on June 27, August 29, and September 17, 2016. Approximately ten bees from each hive were carefully collected in a sterile 50 mL falcon tube from near the brood box entrance. The bees were immediately frozen on dry ice in the field. The samples were brought back to the lab and stored in a -80°C freezer until processing.

### DNA extraction and quantification

DNA was extracted from four Individual Whole Bees from each hive for the months of June, August, and September (n = 240). To reduce sequencing complexity, the four Individual Whole Bee DNA extractions from each hive and at each time point were pooled into one sample in equal concentrations (250 ng DNA from each bee extraction), resulting in 60 Pooled Whole Bee samples. A small subset of the 240 Individual Whole Bees (unpooled, n = 12) were sequenced separately and analyzed to compare with their respective pooled samples.

To compare the microbial community within bee guts to bee bodies and whole bees, DNA was extracted from Individual Bee Guts (n = 10) and the corresponding Individual Gutless Bees (n = 10).

The individual bees used for this comparison were randomly selected from five traditional and five Flow samples. To remove the guts, the head and thorax were first removed leaving only the abdomen. One pair of sterile tweezers held the stinger and one pair held the opposite end of the abdomen. The guts were pulled out from the end with the stinger and placed into a lysing matrix tube for DNA extraction. The remaining head, thorax, and empty abdomen were placed into a second lysing matrix tube (Individual Gutless Bees) for extraction.

DNA extractions were performed using the FastDNA Spin Kit for Soil with Lysing Matrix E tubes (MP Biomedicals, Santa Ana, CA) following the manufacturer protocol (protocol revision number 116560200–201503) with a few minor changes, including: step 5, centrifugation was increased to 10 minutes; step 7, 15 mL tubes were used and rotated at speed 3 for 5 minutes in a ProBlot hybridization oven (Labnet, Edison, NJ); step 9, the tubes were mixed by pipetting rather than shaking; step 12, DES was heated to 55°C and allowed to sit on filter for 2 minutes prior to elution. Bead beating was performed with the FastPrep instrument (MP Biomedicals, Santa Ana, CA) for 40 seconds at a speed setting of 6.0 m/s. DNA was quantified using the Qubit 3.0 Fluorometer (Thermo Fisher, Inc., Waltham, MA). The High Sensitivity dsDNA Assay Kit was used for the majority of the samples, however, some samples with high concentrations of DNA were quantified using the Broad Range dsDNA Assay Kit.

### DNA sequencing

DNA sequencing included: Pooled Whole Bees (n = 60); Individual Whole Bees (unpooled, n = 12); Individual Bee Guts (n = 10) and corresponding Individual Gutless Bees (n = 10); replicate DNA extractions (n = 2) and DNA extraction kit blanks (n = 2) for controls. All samples had a final DNA concentration of 17–40 ng * μL^-1^ with the exception of the DNA extraction kit blanks, which had a concentration of <0.5 ng * μL^-1^. DNA extracts were sent to the DNA Services Facility at the University of Illinois at Chicago Sequencing Core for amplicon 300 bp paired-end sequencing of 16S rRNA genes using the 515F-Y (5’-GTGYCAGCMGCCGCGGTAA) and 926R (5’-CCGYCAATTYMTTTRAGTTT) primers [19–20].

Genomic DNA was PCR amplified with primers CS1_515F and CS2_926R [21] targeting the V4-V5 regions of 16S rRNA genes using a two-stage targeted amplicon sequencing protocol [22–23]. The primers contained 5’ common sequence tags (CS1 and CS2) as described previously [24]. First stage PCR amplifications were performed in 10 microliter reactions in 96-well plates, using the MyTaq HS 2X mastermix. PCR conditions were 95°C for 5 minutes, followed by 28 cycles of 95°C for 30 seconds, 50°C for 60 seconds and 72°C for 90 seconds. Subsequently, a second PCR amplification was performed in 10 microliter reactions in 96-well plates using the MyTaq HS 2X mastermix. Each well received a separate primer pair with a unique 10-base barcode from the Access Array Barcode Library for Illumina (Fluidigm, South San Francisco, CA; Item# 100–4876), which contained the CS1 and CS2 linkers at the 3’ ends of the oligonucleotides. Cycling conditions were as follows: 95°C for 5 minutes, followed by 8 cycles of 95°C for 30 seconds, 60°C for 30 seconds and 72°C for 30 seconds. A final, 7 minute elongation step was performed at 72°C. Samples were pooled in equal volume using an EpMotion5075 liquid handling robot (Eppendorf, Hamburg, Germany). The pooled libraries were subject to size selection (425–525 bp) to remove host DNA amplification products using a Blue Pippin Prep device (Sage Science, Beverly, MA) and employing a 2% agarose gel cassette. The pooled libraries were loaded onto an Illumina MiniSeq mid-output flow cell (2x150 paired-end reads) with a 20% phiX spike in. Based on the distribution of reads per barcode, the amplicons (before purification) were re-pooled to generate a more balanced distribution of reads. The re-pooled libraries were again subject to Pippin Prep size selection under identical conditions. The re-pooled libraries, with a 20% phiX spike-in, were loaded onto a MiSeq v3 flow cell, and sequenced (2x300 paired-end reads) using an Illumina MiSeq sequencer. Fluidigm sequencing primers, targeting the CS1 and CS2 linker regions, were used to initiate sequencing. De-multiplexing of reads was performed on instrument.

### Sequence analyses

Raw paired end sequence reads were processed using QIIME 1.9.1 [25]. Briefly, raw reads were joined with their mate pair based on overlap, then reads were filtered to a Phred quality score of at least 20 where 75% of the read consisted of high-quality bases (>20) and no more than 3 consecutive low-quality bases were encountered. Reads with unassigned “N” base calls were removed. Operational taxonomic units (OTUs) were then picked from the sequences at a 97% identity threshold and taxonomy was assigned using Greengenes release 13_8 [26], as well as manual curation based on the top three BLASTN [27] hits to the SILVA 132 SSU Reference database [28]. Low abundance OTUs that made up <0.005% of all remaining reads were removed from downstream analysis. Chimeric and ‘Indecipherable’ OTUs were detected and removed using the online DECIPHER tool for short-length sequences [29]. All chloroplast and mitochondrial sequences were removed, as well as two OTUs that appeared at similar abundances in all samples including the two DNA extraction kit blank samples.

After processing, 99 bacterial OTUs remained for community analysis. Four of those OTUs were not assigned taxonomy, but were 89–91% identical to 16S rRNA gene sequences in the SILVA 132 SSURef Nr99 database [28]. OTU sequences were aligned and a maximum likelihood phylogenetic tree was assembled using SINA [30].

To correct for variations in sequencing library size for each sample, samples were rarefied to a depth of 20,076 reads. This level of rarefaction excluded 9 samples from further analysis, all of which had fewer than 8,755 total reads. Seven of the removed samples belonged to Individual Gutless Bee samples, one was an Individual Bee Gut sample, and one was a Pooled Whole Bee sample.

The phyloseq R package [31] was used to calculate weighted UniFrac distances between samples [32] and ordinate onto principal coordinates for analysis (PCoA). ANOSIM from the vegan R package [33] was used to calculate differences in community composition between sample groups.

## Results and discussion

### Sample type comparisons

We compared the *A*. *mellifera* microbiome associated with different sample types from the same location: **1)** Pooled Whole Bee DNA extracts combined from four individual bees from the same hive at the same sampling point (n = 59 after data preprocessing, see Methods); **2)** Individual Whole Bee DNA extracts to compare with their respective pooled samples (n = 12); **3)** DNA extracts from dissected Individual Bee Guts (n = 9); and **4)** corresponding Individual Gutless Bees with the remaining bee body after the gut was removed (n = 3). A total of 99 bacterial 16S rRNA gene OTUs were identified across the entire dataset.

Only three Individual Gutless Bee samples were retained after data preprocessing due to systematically low library sizes after read filtering (82–3,020 reads for the seven removed samples). The three remaining samples shared many OTUs (n = 74) with the other sample types, but had inconsistent relative abundance patterns that differed from the rest of the dataset. These inconsistencies and low sample number were cause to remove the Individual Gutless Bee samples from further analyses. Systematically small libraries could have been caused by sparse bacterial populations in the Individual Gutless Bee samples.

Weighted UniFrac distances ordinated onto a PCoA plot showed no clear visual separation based on sample type (S1 Fig). ANOSIM hypothesis tests were used to compare the Individual Bee Gut samples to the Individual Whole Bee samples (R = 0.142, *p* = 0.101), and the Individual Bee Gut samples to the Pooled Whole Bee samples (R = 0.079, *p* = 0.114). The low R values from these comparisons revealed little to no difference in the microbial communities between the dissected Individual Bee Gut samples and the whole bee samples (both Individual and Pooled). This comparison, in addition to the poor-quality libraries from Gutless Bee samples, may indicate that most of the *A*. *mellifera* microbiome sequenced from whole bee samples is derived from the guts. A previous report showed that the *A*. *mellifera*-associated bacterial community structure did not differ between surface sterilized whole bees and unsterilized whole bees [34]. This further suggests that most of the microbial biomass is located inside the insect body relative to its surface. While most *A*. *mellifera* microbiome sequencing efforts have largely focused on the microbial community of the guts [35–37], future efforts targeting the whole bee microbiome may reduce sampling and dissection efforts while still maintaining biologically relevant trends.

To test for the heteroscedasticity of individual bees within a pooled sample, Individual Whole Bees (n = 12) were compared to the corresponding Pooled Whole Bee samples (n = 3). The relative abundance of each OTU in the four Individual Whole Bee samples was compared to values observed in the respective Pooled Whole Bee sample (S2 Fig). Lines were fit to each Individual Whole Bee sample with slopes ranging from 0.731 to 1.095, where a slope of 1 indicated an exact linear relationship between the Individual Whole Bee and Pooled Whole Bee sample. Overall, similar community structure patterns across individual and pooled samples confirmed that Pooled Whole Bees were an adequate representation of each hive at each time point. All subsequent analyses included only the Pooled Whole Bee samples.

### *A*. *mellifera* microbial community structure among hive types

**Fig 1:**
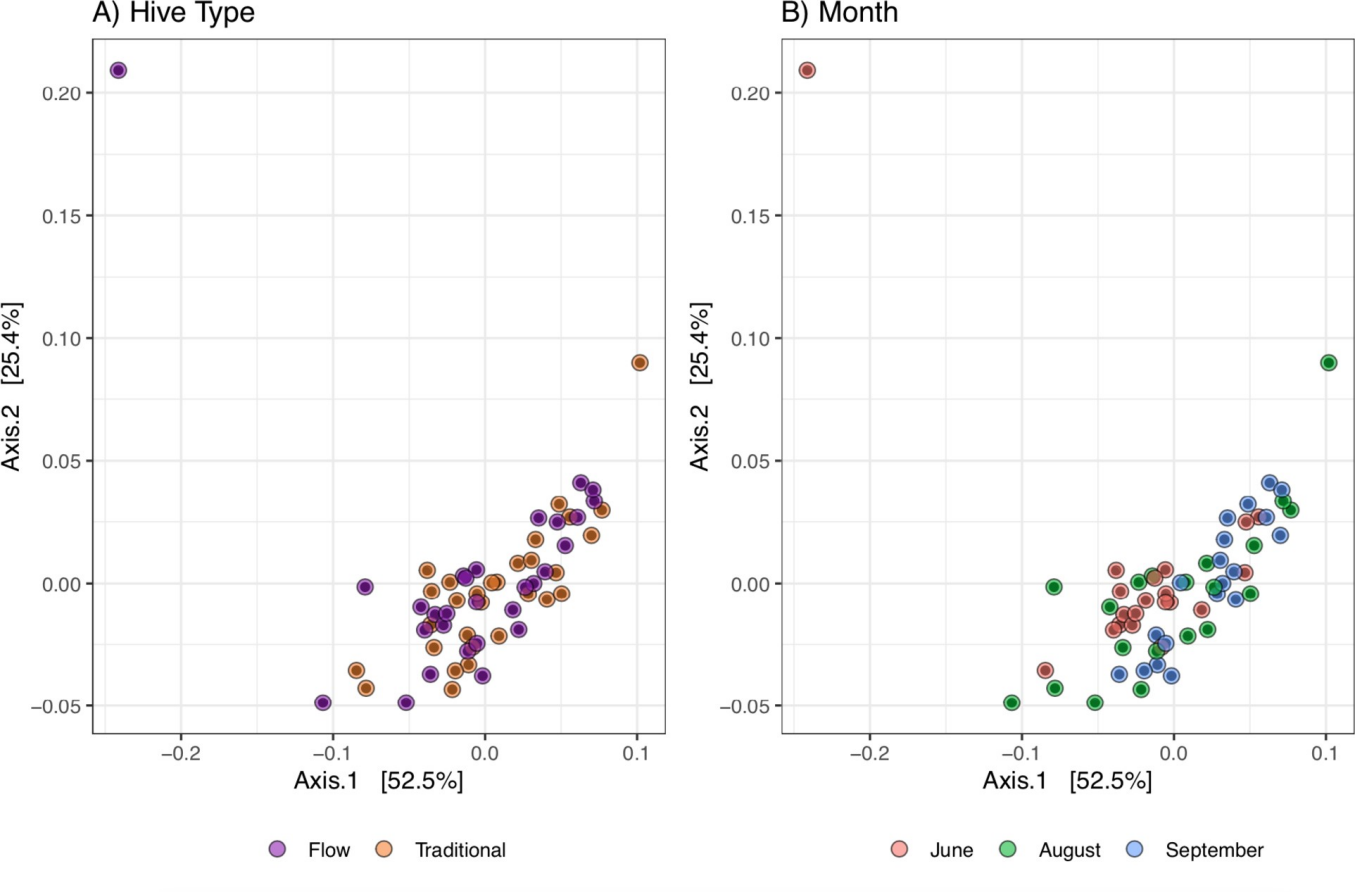
Principal coordinates analysis of the weighted UniFrac distances calculated for the bacterial communities from 59 Pooled Whole Bee samples. Individual samples are colored by **A)** Hive type, or **B)** Sampling month.

**Fig 2:**
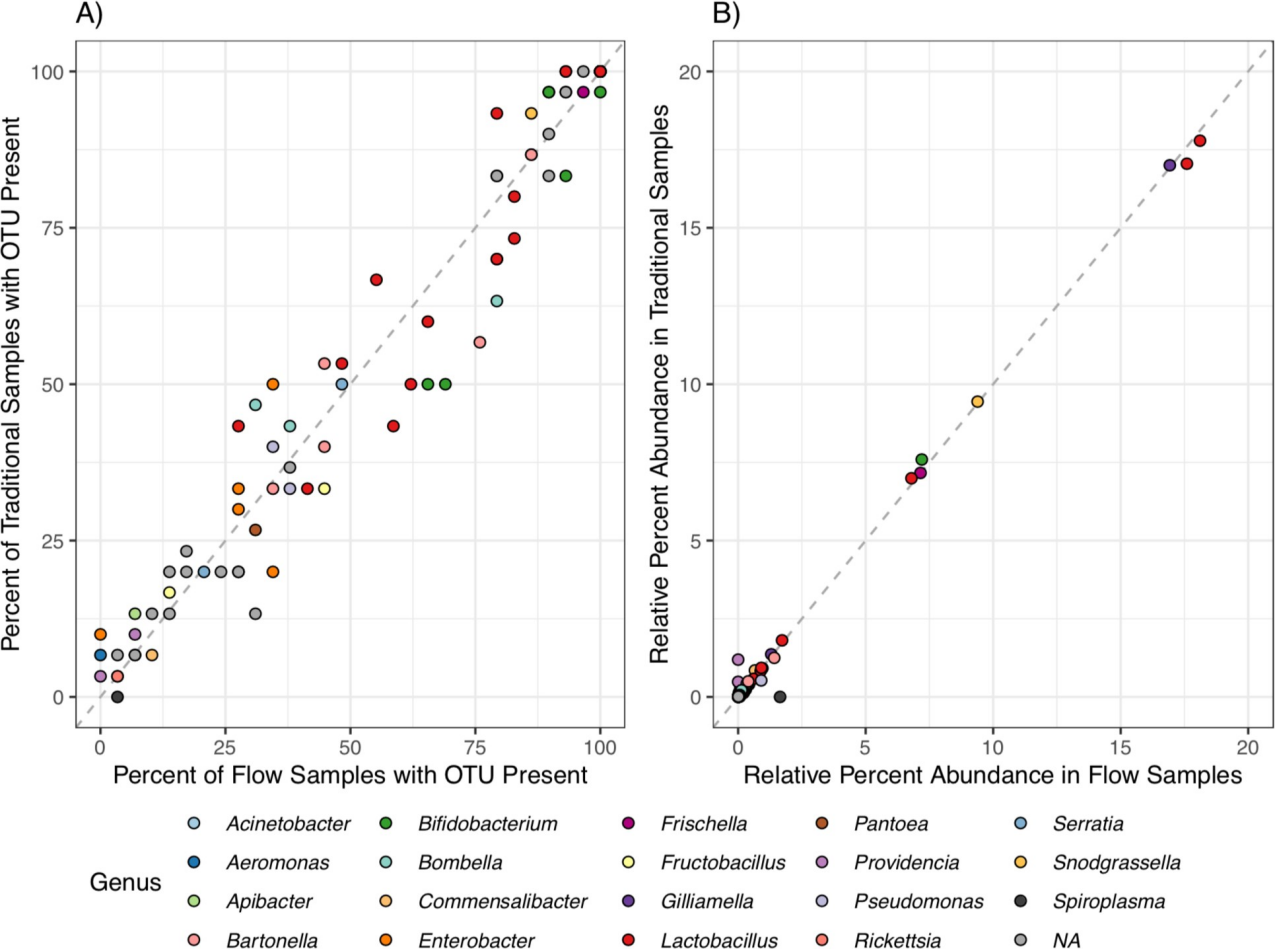
**A)** Comparison of individual OTU presence between Flow and Traditional hive types. The dashed line has a slope of one. **B)** Comparison of individual OTU relative abundance between Flow and Traditional hive types. The dashed line has a slope of one.

No consistent differences in microbial community structure were observed between bees from Flow hives and traditional Langstroth hives based on weighted UniFrac distances (Fig 1). ANOSIM hypothesis tests were run to examine any effects based on hive type overall and separated by month (Table 1). The slightly negative R values indicated that there was more variation with a group than there was between groups. No clear set of OTUs were present in Flow samples but not Traditional samples, and vice versa (Fig 2). The two hive types held very similar relative abundance patterns for each OTU as well (Fig 2). This indicated that even within the low-abundance microbiome, there were no clear differences based on hive type. Overall, the similarities between the microbial communities observed in Flow and Traditional hives indicate that hive construction does not have an immediate effect on bee microbiome; however, long term studies and larger datasets are necessary to evaluate whether hive structure mitigates the risk of stress-induced dysbiosis. Similarities in microbial community structure among hive type may have been influenced by landscape (bees from both hive types foraging on the same landscape), previously shown to impact bee microbiomes [38]. Additional sequence data (e.g., more samples overall, more individual bee samples, and/or greater sequence depth per sample) may have revealed additional patterns among sample types.

**Table 1:** ANOSIM test statistics for comparisons between hive type and month.

Comparison	ANOSIM R Statistic	Significance
Hive Type-All Pooled Whole Bee Samples	-0.031	0.999
Hive Type-June Pooled Whole Bee Samples	-0.067	0.976
Hive Type-August Pooled Whole Bee Samples	-0.073	0.934
Hive Type-September Pooled Whole Bee Samples	-0.085	0.931
Month-All Pooled Whole Bee Samples	0.125	0.002
June vs August Pooled Whole Bee Samples	0.061	0.035
June vs September Pooled Whole Bee Samples	0.247	0.002
August vs September Pooled Whole Bee Samples	0.082	0.03

### *A*. *mellifera* microbial community structure over honey production season

Previous reports showed little seasonal effect on bacterial composition in the total gut of foraging bees [39], and a stable microbiome in the bee midgut/pylorus for hives fed a consistent diet over a four-month period [35]. Here, *A*. *mellifera* microbial communities were evaluated over the honey production season (June, August, and September). There were no clear groupings based on sampling month in the PCoA ordination (Fig 1). ANOSIM hypothesis tests were run to test for differences between all months: while most ANOSIM tests had low *p*-values, the R values were still relatively low (< 0.5), indicating little to no difference in the communities over time (Table 1). Two outlier samples (F17-JUN16 and T5-AUG16; see microbial dysbiosis discussion below) had very small effects on R values (<0.03) of these hypothesis tests.

There was a very small difference between all June samples and all September samples (R = 0.247, *p* = 0.002), which could indicate a slight temporal component to community structure development. The relative abundances of shared OTUs across all June and September Pooled Whole Bee samples revealed decreases in some *Lactobacillus*, *Serratia*, *Enterobacter*, and *Frischella* OTUs and increases in other *Bifidobacterium*, *Lactobacillus*, *Gilliamella*, *Bartonella*, *Pseudomonas*, and *Snodgrassella* OTUs (S3 Fig). A small number of OTUs were found in June samples but not in September samples (n = 9) or vice versa (n = 3); however, none of these unique OTUs were present in all June or September samples, respectively, and most were found at extremely low abundances. Previous reports showed that a small percentage of bacteria found within bee guts were derived directly from pollen [39], so small seasonal changes in the bee gut microbiome may be related to changing pollen sources over time based on flowering patterns of different plants. Similarly, dietary changes over a foraging season were previously associated with changes in midgut/pyloric bacterial community structure: *Gilliamella apicola*, a bacterium that is able to utilize pectin (a main component of pollen) peaked in May when pollen was the primary food source, while *Enterobacteriaceae* increased in September when a sugar mixture was the primary food source [35].

### *A*. *mellifera* core bacterial community

**Fig 3:**
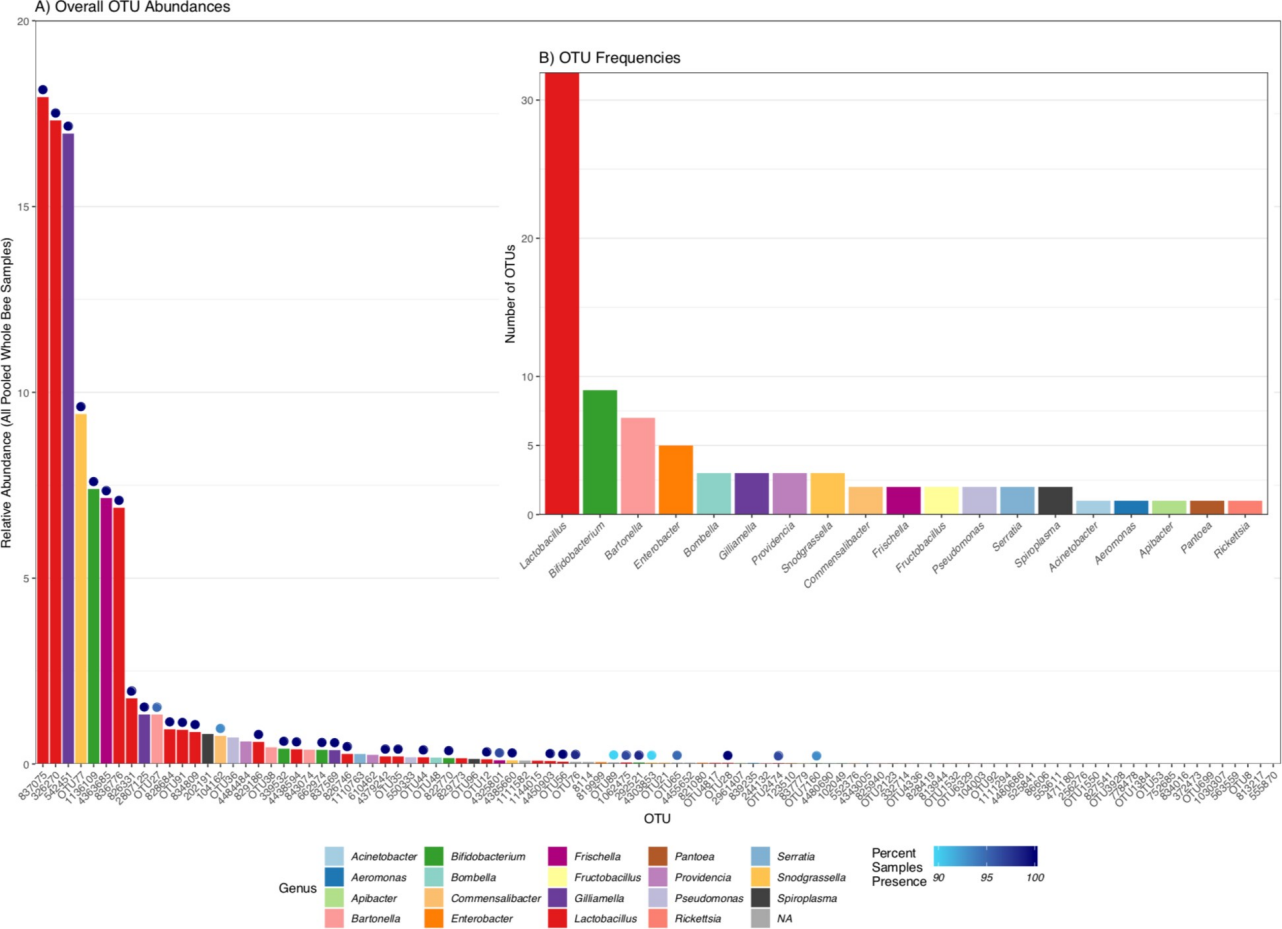
**A)** Distribution of the cumulative relative percent abundance of individual OTUs across 59 Pooled Whole Bee samples colored by genera. Dots above bars represent the percent of conservation (presence/absence) of each OTU within the 59 samples. **B)** Distribution of unique OTUs by genera (83 of the 98 total OTUs were classified to the genus level).

A total of 98 bacterial OTUs were identified in at least one of the 59 Pooled Whole Bee samples. Of those, 82 OTUs were classified at the genus level (with the remaining 16 unclassified) representing only 19 different genera (Fig 3; S4 Fig). The *A*. *mellifera*-associated microbial communities consisted of a highly stable core group of bacterial taxa over time and hive type. The top 10 most abundant taxa made up 87.5% of the rarefied reads from all Pooled Whole Bee samples (Fig 3). Nine of the top ten taxa were found in 100% of all Pooled Whole Bee samples, with the 10th most abundant OTU (OTU27) missing from only three of the 59 samples. Within those top 10 taxa, four OTUs were representatives of the *Lactobacillus* genus, two *Gilliamella*, one *Snodgrassella*, and one *Bifidobacterium*, all of which are commonly associated with the gut microbiome of the honey bee worldwide and are considered part of the core microbiome [17, 40]. Two of the top 10 most abundant OTUs fell within the *Frischella* and *Bartonella* genera. *Frischella perrara* and *Bartonella apis* are commonly associated with the bee gut microbiome, but are not always present and may vary in abundance based on ecological conditions [17, 40].

Across all abundance levels, 39% (38/98) of OTUs were found in ≥ 90% (53/59) of samples, with 27 of those OTUs found in 100% of samples (Fig 3). Thirteen of these highly conserved taxa each contributed ≤ 0.1% of reads to the microbial community (Fig 3). The 13 OTUs were members of the *Lactobacillus* (n = 5), *Bifidobacterium* (n = 2), *Snodgrassella* (n = 2), and *Frischella* (n = 1) genera, while the remaining three OTUs were not confidently assigned taxonomy. The conservation of these low abundance taxa may indicate a strong evolutionary pressure to maintain these organisms, possibly due to environmental constraints, niche differentiation, enzyme kinetics, or competition.

**Fig 4:**
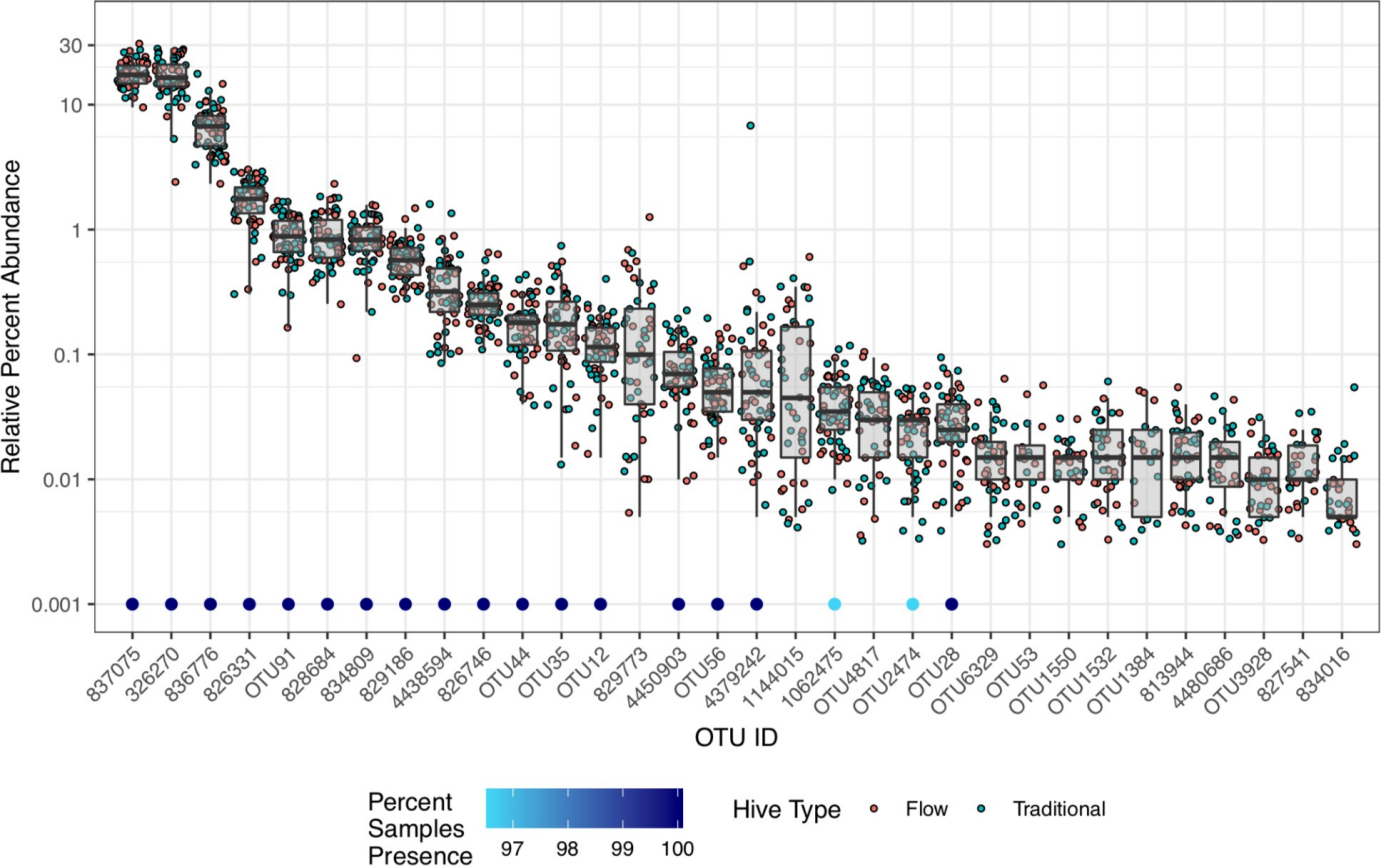
Relative percent abundance of all OTUs belonging to the *Lactobacillus* genus from all 59 Pooled Whole Bee samples. Individual data points represent the relative abundance within one sample colored by hive type. Dots above each OTU name represent the percent of conservation (presence/absence) of each OTU within the 59 samples.

Almost one third of all OTUs (32/98) belonged to the *Lactobacillus* genus, accounting for nearly half (49.2%) of all the Pooled Whole Bee rarefied sequence data with only a few OTUs contributing the majority of reads (Figs 3 and 4). The *Lactobacillus* OTUs ranged from 0.005–17.95% relative abundance, with 18 OTUs found in 100% of Pooled Whole Bee samples (including the top 14 *Lactobacillus* OTUs ranked by median abundance) (Fig 4). The *Lactobacillus* OTUs were equally distributed among hive types.

The second most frequently observed genus was *Bifidobacterium* representing 8.4% of all Pooled Whole Bee rarefied data (Fig 3; S5 Fig). Four of the nine *Bifidobacterium* OTUs were found in 100% of samples. The relative abundance patterns of each *Bifidobacterium* OTU were similar in both hive types.

Several genera with relatively few OTU representatives also dominated the Pooled Whole Bee microbiome. Three *Gilliamella* OTUs accounted for 18.7% of the rarefied sequence data, while three *Snodgrassella* OTUs made up 9.6% and two *Frischella* OTUs made up 7.3%. Of these eight OTUs, six were found in 100% of Pooled Whole Bee samples. A *Frischella* OTU (4325801) was missing from just two samples and a *Snodgrassella* OTU (4303853) was missing from six samples.

These highly conserved taxa indicate a core microbial community within the *A*. *mellifera* gut microbiome, similar to previous studies [17, 40]. The consistent nature of the gut microbiome suggests that these microbes likely have central and necessary functions in bees [17, 40]. *Lactobacillus*, *Bifidobacteria*, and *Gilliamella* spp. play a significant role in modulating bee health in part by mediating pollen degradation (e.g., flavonoids and ω-hydroxy acids in the other pollen wall) and carbohydrate metabolism (e.g., honey and nectar), which produces metabolites that are then utilized by the bee host [41]. Additionally, *Lactobacillus* and *Bifidobacteria* spp. can provide protection against bee pathogens [42, 43]. *Frischella* sp. gain energy from anaerobic fermentation of carbohydrates [44] and has been associated with stimulation of host immune pathways [45, 46]. *Bartonella* sp. ferment carbohydrates under microaerophilic conditions [47] and possesses genes for the degradation of secondary plant metabolites present in pollen and nectar [48]. *Gilliamella* and *Snodgrassella* spp. occupy very different metabolic niches [49] and participate in cross-feeding whereby *Snodgrassella* consumes pyruvate and other metabolites produced by *Gilliamella* [41].

### *A*. *mellifera* auxiliary bacterial community members

While 38 of the OTUs were found in the majority of the Pooled Whole Bee samples, the remaining 60 OTUs were distributed across varying levels of conservation (Fig 3). There were 41 OTUs present in <50% (29/59) of samples ranging in average relative abundance from 0.0003–0.81%. Of the 19 genera identified in this study, 11 were exclusively represented by OTU(s) present in <50% of samples, including *Apibacter*, *Fructobacillus*, and *Pseudomonas*. All five *Enterobacter* OTU representatives were present in <50% of samples and only accounted for 0.1% of all Pooled Whole Bee rarefied data, indicating a potentially transient residency. Three of the observed *Bartonella* OTUs were present in <50% of samples (out of seven total *Bartonella* OTUs that made up 2.2% of all Pooled Whole Bee rarefied data). Additional sequence data (e.g., more samples overall, more individual bee samples, and/or greater sequence depth per sample) may have identified additional patterns in the distribution of auxiliary bacterial OTUs.

### Potential indications of microbial dysbiosis

There were two samples (F17-JUN16 and T5-AUG16) that ordinated further away from most other samples (Fig 1), which may reflect community differences associated with age, physiology, or health status. Both of these samples had high abundances of a few taxa that were not common in other samples. F17-JUN16 had a large influx of two *Spiroplasma* OTUs, which could be a sign of infection. *Spiroplasma* have been shown to opportunistically infect honey bees during the spring season and are thought to cause “May disease,” which a neurological disorder that renders bees unable to fly and can eventually cause death ([50, 51] and references within). Climatic conditions and nectar in flowering plants may impact transmission and infection rates of *Spiroplasma* [50]. Since the *Spiroplasma* OTUs were not observed in August or September samples for hive F17, it is likely that the microorganism was cleared from the hive.

The T5-AUG16 sample had a sharp increase in the abundance of two *Providencia* OTUs. *Providencia* species have been associated with various insects, either from the whole insect or specifically from the gut ([52] and references within), and were recently characterized for the first time in the gut of the *Apis* genus in Saudi Arabia [53]. *Providencia* is pathogenic in the fruit fly (*Drosophila melanogaster*), but pathogenicity varies by species as some cause more fatal infections than others [54].

## Conclusion

Honey bee microbiota are important organisms for maintaining colony health. The new Flow frame system promotes a change in the way a beekeeper produces honey and manages bees with the intent of decreasing colony stress through less disturbance. We examined if different hive technologies, and specifically the way honey is harvested from those technologies, influenced the microbiome of the bees. This study did not find a difference in the *A*. *mellifera*-associated microbial communities based on hive or sample type, but a small difference was observed between June and September indicating a temporal influence on community structure that may be related to changing forage during the sample period. The highly abundant taxa observed in the bee microbiome are derived from the gut indicating that the majority of the bacterial community is associated with the gut rather than the body of the honey bee. Future studies in this area could monitor different stress levels (i.e., movement of hives long distances or along urban or resource gradients) to determine if dominant microbiome communities persist.

## Supporting information

S1 Fig Principal coordinates analysis of the weighted UniFrac distances calculated for the microbial communities from 83 *A*. *mellifera* samples of varying sample types and hive constructions. (PDF)

S2 Fig Direct comparisons of the relative percent abundance of all OTUs in Individual Whole Bee samples to their respective Pooled Whole Bee sample. Lines were fit for each Individual Whole Bee Sample and displayed in a table. Dashed lines have a slope of one, representing an exact correlation. Each individual sample within a hive was color coded (Red = bee 1, Blue = bee 2, Green = bee 3, Yellow = bee 4).
(PDF)

S3 Fig Changes in relative percent abundance between 83 shared OTUs from June to September. All observations of a given OTU were summed in all June samples and all September samples, respectively. Differences in abundance from June to September are presented as **A)** absolute changes in relative percent abundance (September—June), and **B)** log_2_ fold change (log2(September/June)).
(PDF)

S4 Fig Rarefied abundances of 99 OTUs across 59 Pooled Whole Bee samples, colored by genus. All samples were rarefied to 20,076 reads. Sample names correspond to hive type (T, Traditional or F, Flow), number (1–20), and sampling date.
(PDF)

S5 Fig Relative percent abundance of all OTUs belonging to the *Bifidobacterium* genus from all 59 Pooled Whole Bee samples. Individual data points represent the relative abundance within one sample colored by hive type. Dots above each OTU name represent the percent of conservation (presence/absence) of each OTU within the 59 samples.
(PDF)
