# Supplementary figures and images for: Honey bee microbiome associated with different hive and sample types over a honey production season
Source: PLoS One. 2019 Nov 8;14(11):e0223834. doi: 10.1371/journal.pone.0223834 (PMC6839897; doi:10.1371/journal.pone.0223834)

### Figure S1

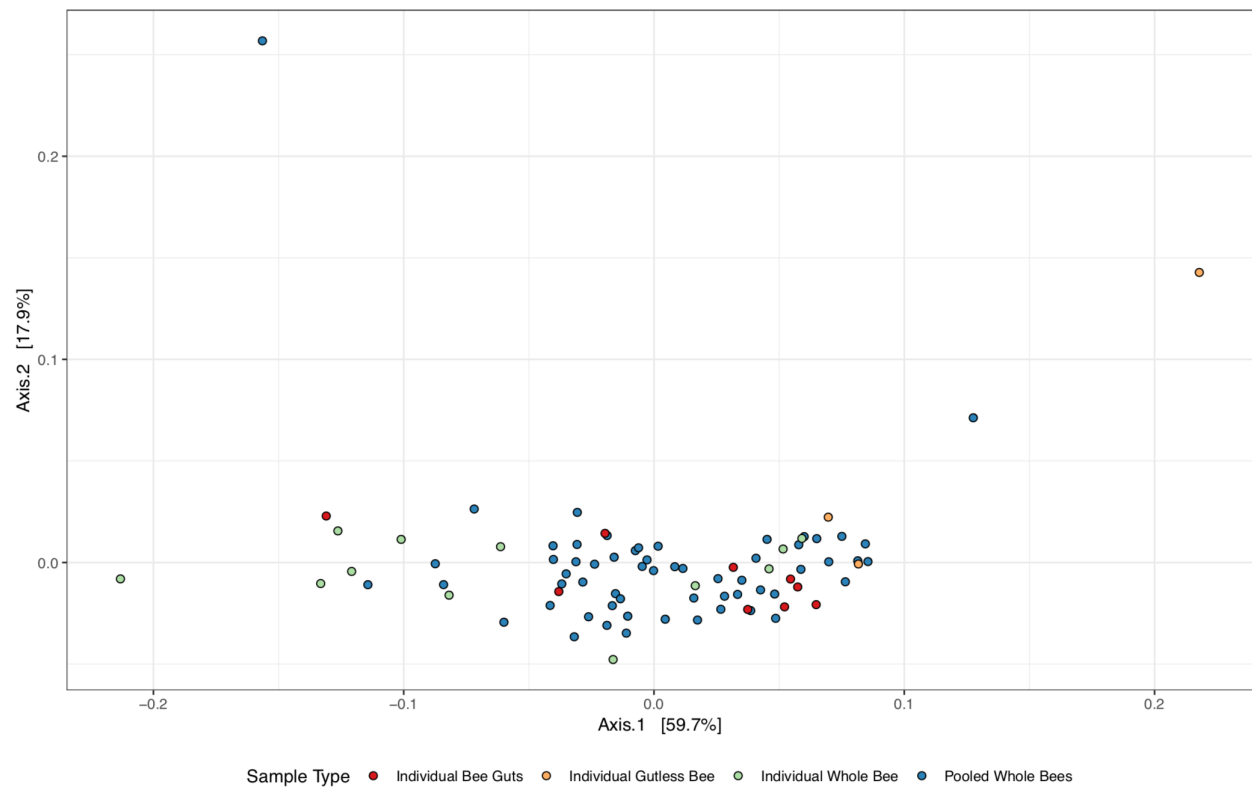

Supplement: S1 Fig — (PDF) [file pone.0223834.s001.pdf]

Figure S2

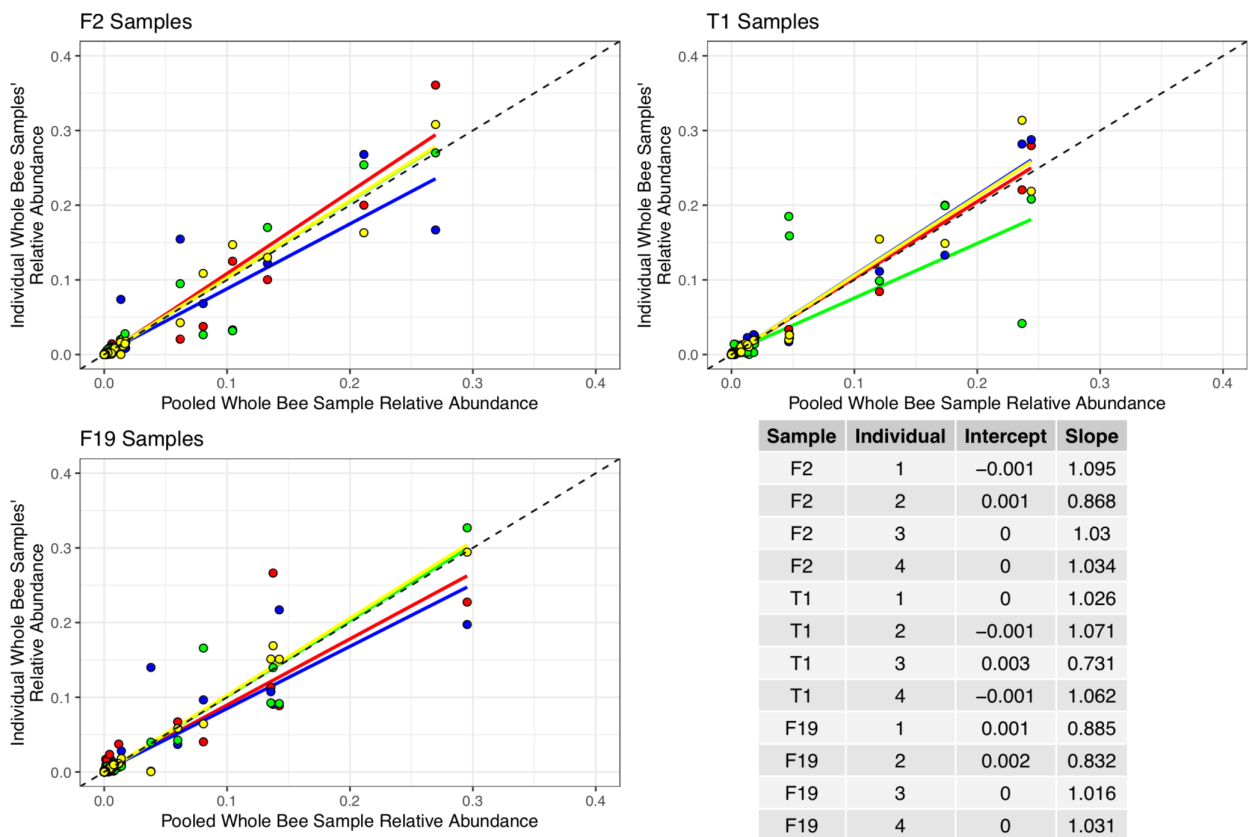

Supplement: S2 Fig — Lines were fit for each Individual Whole Bee Sample and displayed in a table. Dashed lines have a slope of one, representing an exact correlation. Each individual sample within a hive was color coded (Red = bee 1, Blue = bee 2, Green = bee 3, Yellow = bee 4). (PDF) [file pone.0223834.s002.pdf]

Figure S3

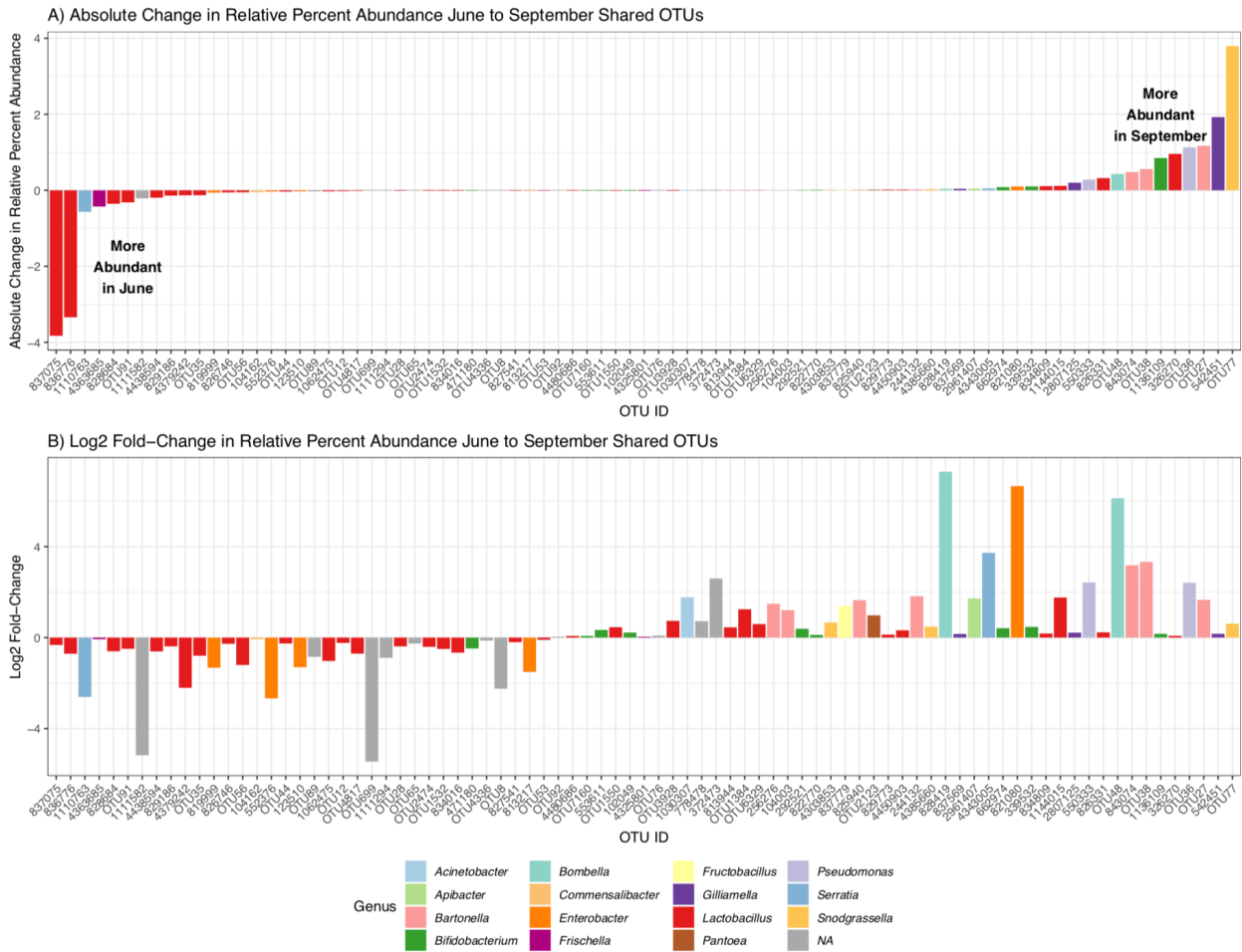

Supplement: S3 Fig — All observations of a given OTU were summed in all June samples and all September samples, respectively. Differences in abundance from June to September are presented as A) absolute changes in relative percent abundance (September—June), and B) log2 fold change (log2(September/June)). (PDF) [file pone.0223834.s003.pdf]

Figure S4

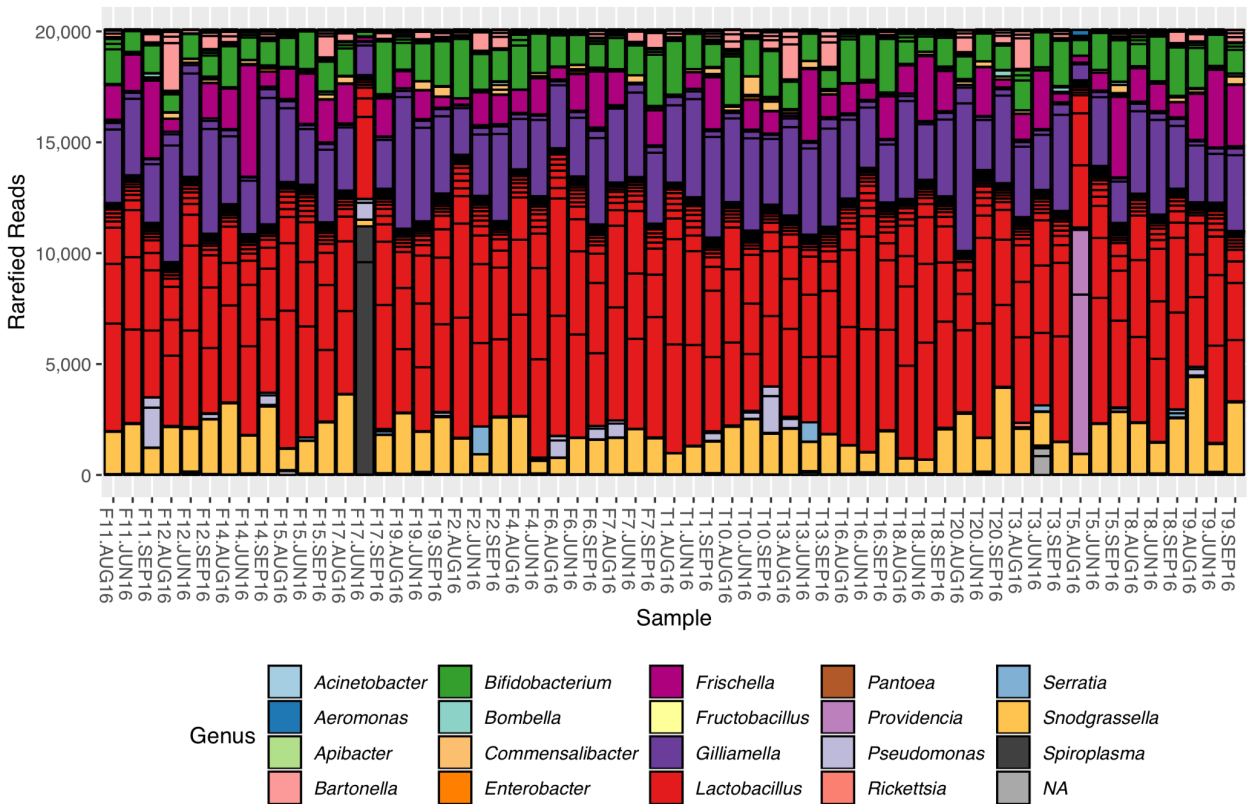

Supplement: S4 Fig — All samples were rarefied to 20,076 reads. Sample names correspond to hive type (T, Traditional or F, Flow), number (1–20), and sampling date. (PDF) [file pone.0223834.s004.pdf]

Figure S5

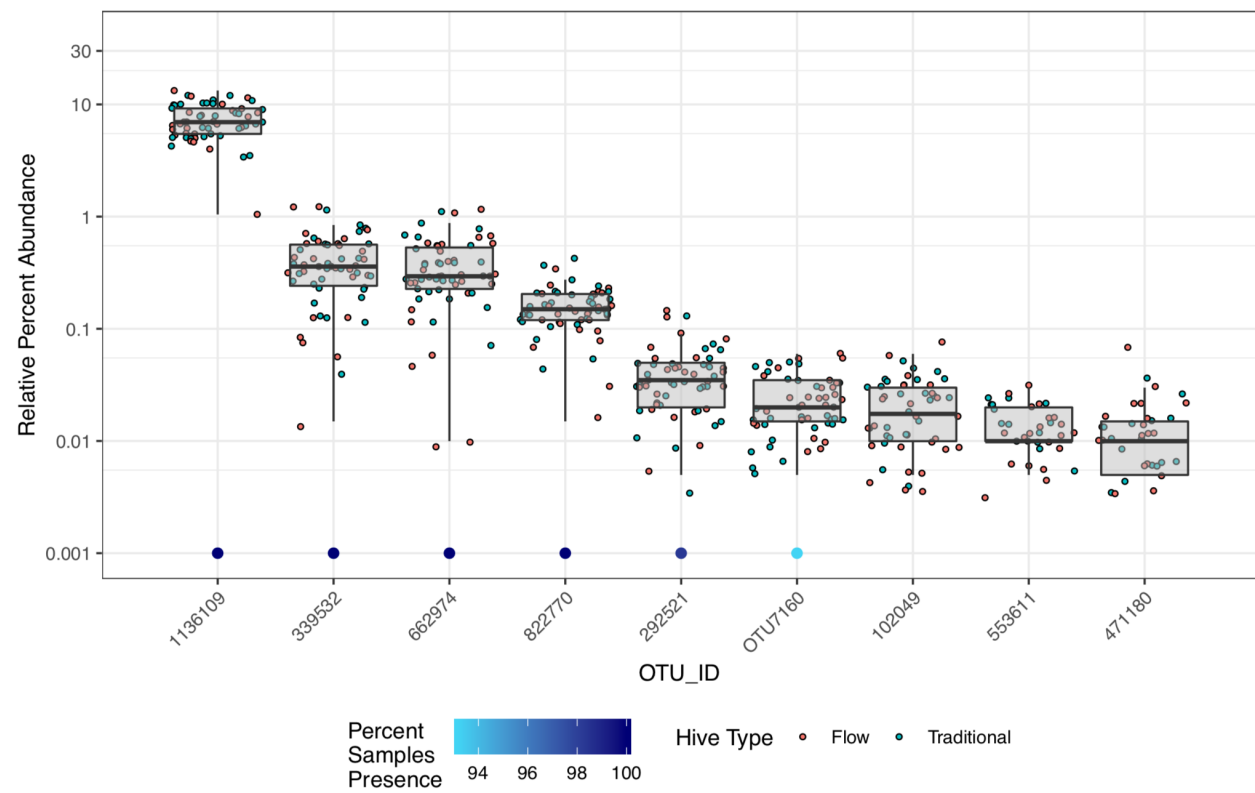

Supplement: S5 Fig — Individual data points represent the relative abundance within one sample colored by hive type. Dots above each OTU name represent the percent of conservation (presence/absence) of each OTU within the 59 samples. (PDF) [file pone.0223834.s005.pdf]
